# Mystery machine: the complex roles of NLRX1 in viral infection

**DOI:** 10.3389/fimmu.2025.1581313

**Published:** 2025-04-28

**Authors:** Mackenzie K. Woolls, Carley M. Elliott, Hannah M. Ivester, Irving Coy Allen

**Affiliations:** ^1^ Graduate Program in Translational Biology, Medicine, and Health, Virginia Tech, Roanoke, VA, United States; ^2^ Biomedical and Veterinary Sciences Graduate Program, Virginia Maryland College of Veterinary Medicine, Virginia Tech, Blacksburg, VA, United States; ^3^ Department of Basic Science Education, Virginia Tech Carilion School of Medicine, Roanoke, VA, United States; ^4^ The Virginia Tech Center for Emerging, Zoonotic and Arthropod-borne Pathogens, Virginia Tech, Blacksburg, VA, United States

**Keywords:** interferons, MAVS, PAMP, regulatory, antiviral

## Abstract

Effective antiviral immunity requires a delicate balance between controlling infection and preventing excessive inflammation. NLRX1, an atypical member of the NOD-like receptor family, plays a crucial regulatory role in this process by modulating immune responses to both RNA and DNA viruses. Unlike other NLRs, NLRX1 does not directly activate inflammatory pathways, but rather fine tunes immune responses through interactions with key signaling initiators like MAVS, FAF1, viral RNA, and FBXO6. These interactions allow NLRX1 to influence antiviral pathways in a highly context-dependent manner. In RNA virus infections, NLRX1 can either enhance immune signaling to restrict viral replication or suppress type 1 IFN responses to promote viral persistence. Similarly, in DNA viral infections, NLRX1 exerts either protective or pathogenic effects, though the precise mechanisms remain unclear. Emerging evidence suggests that NLRX1 may also serve as a key regulator of inflammation and metabolic processes during infection, further contributing to its complex role in immunity. By synthesizing current research, this review provides insight into how NLRX1 regulates immune signaling in RNA and DNA viral infections, highlighting its dynamic role in antiviral immunity and the remaining gaps in our understanding.

## Introduction

**Figure 1:**
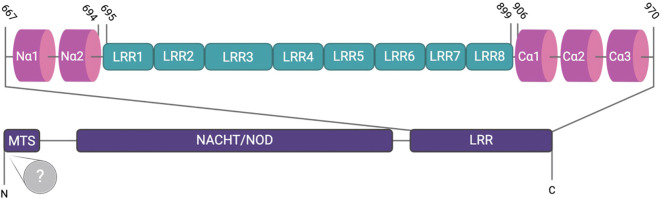
The structure of NLRX1. NLRX1 contains an N-terminal mitochondrial targeting sequence (MTS) responsible for mitochondrial localization, followed by the NACHT/NOD domain and subsequent leucine-rich repeats (LRRs 1-8), which are involved in ligand recognition. The N-terminus consists of two α-helices, whereas the C-terminus, which harbors three α-helices, facilitates dimerization. Despite identification of the MTS, the N-terminus remains largely undefined.

NLRX1 is a member of the Nod-like receptor family and has been implicated in a variety of immune regulatory processes, although its true function remains highly nuanced and appears to be cell type and context specific. Unlike other cytosolic NLRs, NLRX1 has a mitochondrial translocation sequence and can localize to the mitochondria but has also been found in the cytosol (1). NLRX1 negatively regulates multiple inflammatory signaling pathways, including RIG-I and NF-κB signaling, during viral infections to facilitate inflammation resolution and maintain immune system homeostasis (2). In addition to regulating antiviral and anti-inflammatory signaling mechanisms, NLRX1 has also been increasingly recognized for its role in regulating reactive oxygen species (ROS) production and metabolic inflammation (2, 3). Structurally, NLRX1 is comprised of a N-terminal α-helical domain (NLRRNT), a mitochondrial targeting sequence (MTS), a nucleotide-binding and oligomerization (NACHT) domain, eight central LRR modules (LRRM), and three helix C-terminal fascicle (LRRCT) (4) (**Figure 1**). The C-terminal of NLRX1 is responsible for the direct binding of ssRNA and dsRNA (**Figure 1**). Like other pattern recognition receptors, NLRX1 functions through the formation of multi-protein complexes, with cellular effects dictated by the specific interactions and protein scaffolding components that interact with the C-terminal end (**Figure 1**) (4). In contrast to other NLR family members, the N-terminus of NLRX1 remains poorly characterized, with the mitochondrial targeting sequence being the only well-defined region (**Figure 1**) (4). NLRX1 remains a highly enigmatic member of the NLR family. In this review we will discuss the multifaceted role NLRX1 plays in modulating the fine-tuned host immune response during viral infection.

## RNA viruses

Early studies of NLRX1 focused on identifying its role in regulating ssRNA viruses. Pivotal work in the early 2000s identified NLRX1 as a negative regulator of type I interferon in response to ssRNA stimulation. Following infection with a variety of viruses, including simian virus 5 (SV5), sendai virus (SeV), vesicular stomatitis virus (VSV), H1N1 influenza A virus, and the RNA analog poly (I:C), a significant increase in IFN-β production was observed in *Nlrx1^-/-^* mouse embryonic fibroblasts (MEFs) and in mice that was not observed at basal levels. *Nlrx1* ^-/-^ mice infected with sublethal influenza A virus also showed an increase in *Ifnb1*, *Ifna2*, *Oas1a* and *Stat2* transcription levels in lung samples (2). This work ultimately concluded that NLRX1 negatively regulates MAVS function through interactions with RIG-I and attenuates NF-κB signaling through interactions with TRAF proteins (2). However, the full mechanism associated with NLRX1 attenuation of inflammation following influenza virus infection is still unclear. For example, a third study revealed that alveolar macrophages produced significantly lower type-I IFN mRNA transcripts upon infection with A/PR/8/34 (5). This study also suggested that antiviral gene expression was similar between lung homogenates of wild-type and *Nlrx1^-/-^* mice following infection with influenza A virus and extended these findings to include *Nlrx1^-/-^* bone marrow derived macrophages (BMDMs) and MEFs infected with SeV (6). Many of the conflicting findings in the field may stem from differences in the reported subcellular localization of NLRX1, with some studies indicating its presence on the cytosolic surface of the mitochondria (6), while others report localization within the mitochondrial matrix (7). Notably, certain studies suggest that NLRX1 interacts with proteins within the mitochondria rather than with outer membrane-associated proteins such as MAVS (7). This variation underscores a critical gap in the literature concerning the precise localization of NLRX1 and its implications for mitochondrial signaling pathways.

**Figure 2:**
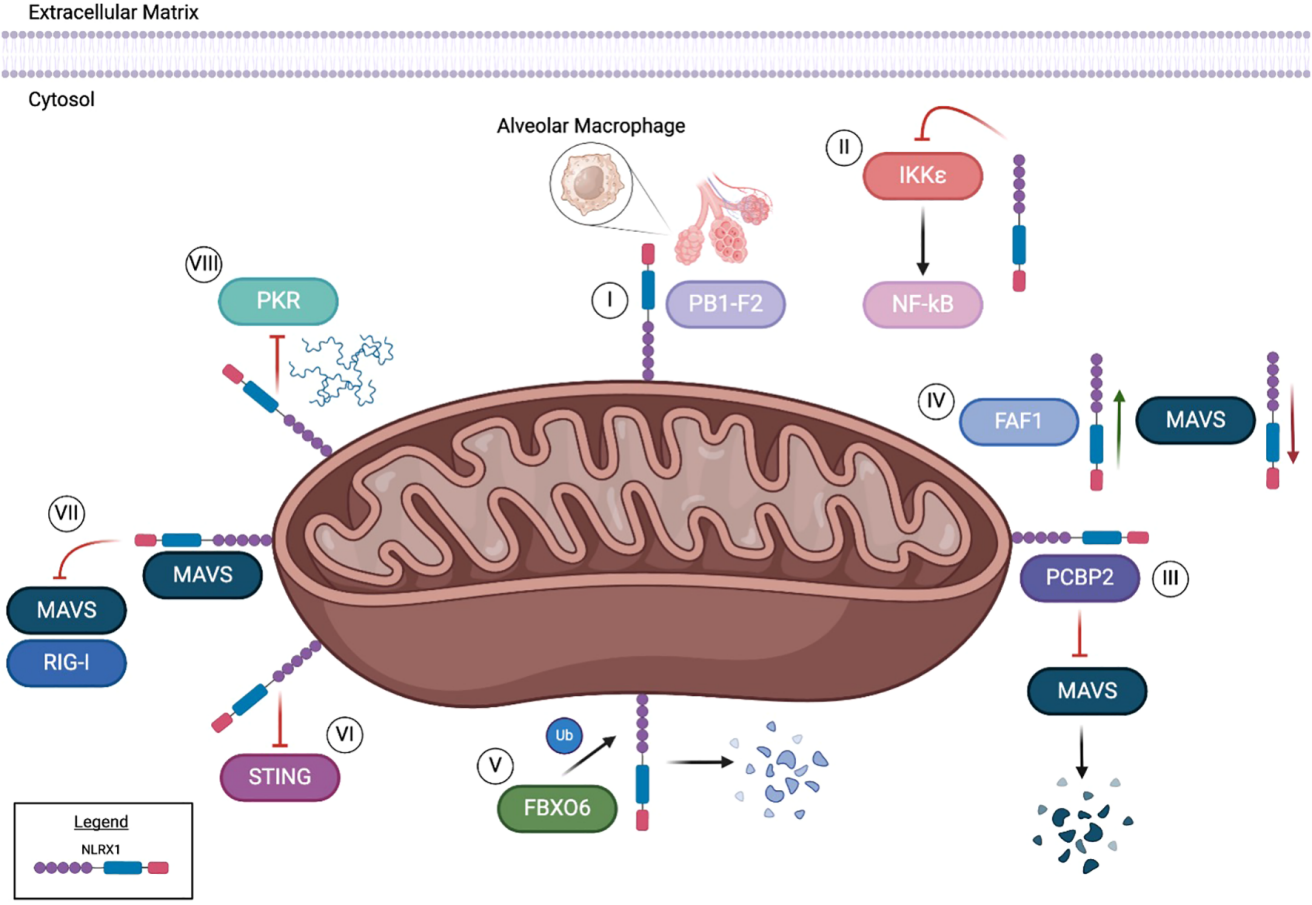
NLRX1 executes a variety of functions from its location in and around the mitochondria. NLRX1 binds to viral protein PB1-F2, ultimately preventing influenza A induced alveolar macrophage damage and apoptosis [I]. When challenged with HIV, NLRX1 has been shown to inhibit IKKϵ, subsequently leading to the downstream inhibition of NF-kB [II]. PCBP2 and NLRX1 have been reported to bind through the NLRX1 NOD region in certain conditions. This binding results in the inhibition of MAVS, ultimately inducing ubiquitination [III]. However, FAF1 is hypothesized to competitively bind with NLRX1, consequently limiting interplay between MAVS and NLRX1 [IV]. An E3 ubiquitin ligase, FBXO6, has been reported to interact with NLRX1, prompting degradation through K48-linked polyubiquitination [V]. In the context of HEK293T cells, NLRX1 inhibits the STING pathway in both endogenous and overexpression models [VI]. DNA viral infections can be modulated through NLRX1 regulation of the MAVS/RIG-I pathway [VII]. Through binding with NLRX1, MAVS interaction with RIG-I is decreased during Hepatitis B viral infections. In ssRNA viruses such as SEV and HAV, NLRX1 competitively binds to viral dsRNA to inhibit PKR pathway activation [VIII].

Consistent with the other members of the NLR family, NLRX1 also functions through forming multi-protein complexes inside the cell following stimulation. Its best characterized functions are associated with the negative regulation of signaling pathways driven by other pattern recognition receptors. As mentioned above, NLRX1 interacts with RIG-I and MAVS to inhibit type I interferon signaling and TRAF proteins following TLR activation to attenuate NF-κB signaling. However, more recently NLRX1 has been shown to also regulate apoptosis through mechanisms that are independent of RIG-I/MAVS and NF-κB following infection with influenza virus. Like its interactions with TRAF proteins, NLRX1 also interacts with the E3 ubiquitin ligase FBXO6 (1). FBXO6 induces apoptosis following infection with influenza virus (8). In HEK293T cells, as levels of FBXO6 increase, the protein levels of NLRX1 decrease in tandem (1). Coimmunoprecipitation revealed FBXO6 and NLRX1 interact together in the cytosol (**Figure 2**) (1). It should be noted that these studies were based on dual overexpression conditions. Subsequent studies confirmed these findings and revealed that FBXO6 degradation of NLRX1 was attenuated upon treatment with MG123, a proteasome inhibitor (1). This study suggests that FBXO6 is responsible for the post-transcriptional decrease in NLRX1 and further demonstrates that the degradation of NLRX1 was achieved through K48-linked polyubiquitination that ultimately resulted in apoptosis (**Figure 2**) (1).

In addition to interactions with host proteins, NLRX1 has also been found to interact with the influenza virulence factor PB1-F2 (5). PB1-F2 influences the virulence of influenza A virus in a strain and host specific manner through modulating apoptotic cell death, type I interferon response, and the activation of the NLR inflammasome (8, 9). Intriguingly, these are also all functions regulated by NRLX1. NLRX1 and PB1-F2 interactions were confirmed using both confocal microscopy and coimmunoprecipitation following influenza infection in HEK293T cells and human airway epithelial (A549) cells (**Figure 2**) (5). This interaction was disrupted when cells infected with influenza A virus were devoid of PB1-F2. In alveolar macrophages, the interaction of NLRX1 with PB1-F2 was observed to be localized to the mitochondria following virus infection (5). This interaction within macrophages was associated with enhanced type I IFN secretion and improved antiviral responses in the host (5).

Many of the influenza studies have been paralleled with other ssRNA viruses, including Sendai virus (SeV) and hepatitis C virus (HCV). Similar to the findings for influenza virus, BMDMs and mouse embryonic fibroblasts from *Nlrx1^-/-^* mice have increased expression of *Ifn-β, Tnf* and *Il-6* mRNA in response to SeV infection. Modulation of IFN responses is also predicted to occur through NLRX1 negative regulation of MAVS/RIG-I and NF-κB following SeV infection, vesicular stomatitis virus (VSV), and encephalomyocarditis virus (EMCV) infection (2). These findings have also been observed in RAW264.7 cells, where NLRX1 has been shown to competitively bind to FAS-associated factor 1 (FAF1) following influenza and VSV (10). The interaction with FAF1 has also been observed in HEK293T cells and BMDMs, which appears to disrupt the binding of NLRX1 with MAVS (**Figure 2**) (10). As interactions between FAF1 and NLRX1 increase, interactions between MAVS and NLRX1 decrease (10). These findings suggest that FAF1 competitively binds to NLRX1, allowing the activation of the MAVS/RIG-I pathway and induction of interferons to drive the host response to influenza infection.

In addition to the regulation of NLRX1 function by FAF1 during influenza infection, NLRX1 driven antiviral signaling can also be attributed to the attenuation of viral protein translation. In human hepatocyte cells (PH5CH8), NLRX1 knockout through CRISPR resulted in decreased levels of IRF1 protein, but not *IRF1* mRNA (11). While the levels of IRF1 protein were reduced in the absence of NLRX1, its half-life remained comparable to that in wild-type cells, suggesting that NLRX1 protects IRF1 from SeV-induced inhibition of protein synthesis (11). The loss of NLRX1 also resulted in increased SeV induced activation of PKR and phosphorylation of eIF2α. When both NLRX1 and PKR were deleted, protein synthesis of IRF1 was restored (11). Based on findings from both endogenous and overexpression studies, regulation of IRF1 did not involve direct interactions between NLRX1 and PKR (11). However, overexpression of NLRX1 decreased binding between PKR and viral RNA. This suggests that NLRX1 inhibits SEV induced translational inhibition by competitively binding to viral RNA to decrease the activation of the PKR pathway (**Figure 2**) (11). It should also be noted that this mechanism in PH5CH8 cells appears to be specific for the regulation of IFN-I signaling, as IL-6 mRNA levels were decreased in the absence of NLRX1, suggesting that NF-κB signaling is reduced in this model (11).

NLRX1 also plays a critical role in regulating the host immune response to hepatis C virus (HCV). As with the other RNA viruses discussed above, in a human hepatoma cell line (HLCZO1), HCV infection results in the upregulation of *NLRX1* expression (12). This increase in NLRX1 coincides with a decrease in viral replication and increase in the expression of *IFN-β, IL-28A*, and IFN-stimulated genes (12). When NLRX1 is overexpressed in this system, the inverse results were observed following HCV infection (12). However, these effects appear to be cell type and virus specific, as similar studies in PH55CH8 cells infected with either HCV or hepatitis A virus (HAV) demonstrated increased virus replication (11). Additional differences in these findings could be due to variations in the method of infection. The studies conducted with PH5CH8 cells utilized electroporation of synthetic viral RNA and microRNA mir122 (12), whereas the studies in HLCZO1 utilized natural virus infection (11). Consistent with the role of NLRX1 discussed for other RNA viruses, the mechanism of NLRX1 function during HCV infection is through negative regulation of MAVS (12). Here, NLRX1 was suggested to attenuate MAVS through degradation and regulates phosphorylation of TBK1 and STAT1 (12). NLRX1 coimmunoprecipitated with MAVS during HCV infection (**Figure 2**) (12). Increasing levels of NLRX1 resulted in increased MAVS degradation, which was also observed using a MAVS mutant that was resistant to HCV protease degradation (12). Attenuation of MAVS degradation was observed following treatment of HLCZO1 cells with the proteasome inhibitor MG132 and upon NLRX1 knockdown (12). Degradation was dependent on the NOD region of NLRX1 (**Figure 1**) (12). Overexpression of the Poly(rC)-binding protein 2 (PCBP2), a cellular protein that binds the HCV genome, increased the degradation of MAVS, further suggesting that degradation was directly associated with the NLRX1/PCBP2 interaction (**Figure 2**) (12).

## DNA viruses

NLRX1 was originally defined as a critical regulator of the host immune response following ssRNA virus infection. As discussed above, NLRX1 modulates the activity of other pattern recognition receptors, specifically RIG-I and TLRs, and is also able to directly bind the viral RNA (**Figure 2**). However, more recent studies have expanded its role in regulating the host-pathogen interactions following infection with DNA viruses. No studies have shown direct binding or interactions with viral DNA. Rather, NLRX1 appears to regulate pattern recognition receptor signaling associated with cGAS and STING (13). Unlike the modulation of the host immune response to RNA viruses, NLRX1 plays a more nuanced role following DNA virus infection. For example, following human immunodeficiency virus (HIV) infection in human monocyte cells (THP-1), deletion of NLRX1 using CRISPR editing and shRNA resulted in decreased p55, a HIV Gag protein, as well as reduced virus replication (13). These findings were replicated in human patient derived macrophages and dendritic cells following NLRX1 knockdown using siRNA, indicating HIV replication and nuclear import of HIV-1 DNA was inhibited (13). The decrease in HIV replication corresponded with an increase in IFN-β (13). *NLRX1^-/-^* THP-1 cells treated with an IFN-α/β antagonist resulted in the levels of HIV replication returning to those observed in the control unmodified THP-1 cells (13). Treatment with nevirapine, which blocks HIV-1 complementary DNA synthesis, resulted in an increase of *IFNβ, IL6*, and *RANTES*, while *ISG15* was attenuated (13). These data suggest that NLRX1 negatively regulates the immune response, potentially through the inhibition of NF-κB signaling, as HIV-1 is reverse-transcribed from DNA (**Figure 2**). Following infection with a vaccine strain of HIV (HIV-VSV), TBK1, IKKϵ, IRF3, and STAT1 were increasingly phosphorylated compared to wild-type cells. Together, these data indicate that NLRX1 regulates IFN signaling to attenuate HIV-1 replication, albeit through a mechanism that is not likely related to MAVS signaling. However, regulation of gene transcription via NF-κB signaling may be through mechanisms similar to those reported for the RNA viruses.

STING is an essential sensor in the recognition of the HIV-1 virus. Overexpression of NLRX1 in HEK293T cells reduce STING-induced activation of an IFN-stimulated responsive element (ISRE) luciferase reporter following HIV-1 infection. Additionally, a cGAMP challenge known to activate STING resulted in increased transcription levels of *IFNB, IL6* and *TNF* in the absence of NLRX1. In MEFs from *Nlrx1^-/-^* mice, increased levels of phosphorylated TBK1 were observed following virus infection, which was shown to be associated with NLRX1 inhibition of the STING pathway (**Figure 2**). These findings were corroborated in human THP-1 cells, where both endogenous and overexpressed NLRX1 exhibited enhanced interaction with STING, demonstrated by co-immunoprecipitation, following HIV-1 challenge relative to uninfected controls. Spatially, confocal microscopy and coimmunoprecipitation of mitochondria associated membrane proteins further revealed that the interaction between STING and NLRX1 takes place in the mitochondrial outer membrane (**Figure 2**) (13). In human patient specimens, NLRX1 is down regulated in peripheral blood mononuclear cells taken from HIV+ individuals, with unknown clinical implications to date (14).

In addition to HIV-1, NLRX1 has also been evaluated in the host response to Hepatitis B virus (HBV). In studies using HepG2 and Huh7 cells, silencing NLRX1 resulted in reduced levels of HBV DNA, indicating attenuated replication. Conversely, in cells transduced to overexpress NA+/taurocholate Co-transporting polypeptide (NTCP) and NLRX1, HBV DNA and RNA was significantly increased (15). As with other viruses, levels of *IFN-α, IFN-β*, and *IL-6* increased during HBV infection in HepG2-NTCP cells when NLRX1 was silenced, whereas NLRX1 overexpression decreased the expression of these inflammatory mediators (15). As discussed above in the context of HIV-I, NLRX1 functions through interactions with STING. However, for HBV, NLRX1 regulates the type I interferon response through interactions with MAVS, similar to the mechanisms reported for the RNA viruses. In HepG2-NTCP and Huh7-NTCP cells, NLRX1 and MAVS bind to attenuate MAVS/RIG-I signaling during HBV infection (**Figure 2**). Conversely, when NLRX1 is silenced, the interaction between MAVs and RIG-I is increased (15). These data were confirmed in cells that overexpress a mutant transcript of NLRX1 (p.Arg707Cys) (MT NLRX1) (16), which is a variant commonly found in patients with HBV infection (17).

NLRX1 also negatively regulates inflammation during infection with Kaposi’s sarcoma-associated herpes virus (KSHV). In KSHV latently infected epithelial cells (iSLK.219) and a lymphoma cell line (BCLB-1) cells, siRNA driven NLRX1 knockdown resulted in attenuated lytic replication and reduced KSHV genome copy number (18). Whole genome transcriptional profiling revealed NLRX1 knockdown suppressed and slowed KSHV gene expression in both cell lines (18). Consistent with the response observed for other viruses, the *NLRX1^-/-^* cells had increased *IFNβ* and other JAK/STAT related gene transcripts (18). Likewise, when NLRX1 was overexpressed, IFNβ signaling was attenuated, confirming its role in negatively regulating the interferon response (18). The mechanism of IFN regulation is dependent on TBK1. When *NLRX1^-/-^* iSLK.219 cells were treated with a TBK1 inhibitor, BX795, *IFNβ* levels were diminished down to wild-type levels (18). Consistent with the data for other viruses, NLRX1 was also shown to negatively regulate MAVS during KSHV infection to further regulate the type I interferon response (**Figure 2**) (18).

## Concluding remarks

While significant progress has been made in understanding the function of NLRX1 and other members of the novel regulatory NLR sub-group, many questions remain unanswered, such as the role of NLRX1 on regulation of host metabolism during infection, and the mechanism behind which NLRX1 recognizes and responds to DNA viruses. Like other pattern recognition receptors, NLRX1 functions through forming multi-protein complexes. It also functions through the regulation of signaling associated with the activation of other pattern recognition receptors, including TLRs, NLRs, and Rig-I like helicase receptors. There are many overlapping mechanisms regulated by NLRX1, such as the attenuation of MAVS signaling, shared between the host responses to both RNA and DNA viruses. However, it is also clear that there are multiple unique cell types and temporal specific signaling mechanisms that are significantly less characterized. These mechanisms appear to be driven by unique interactions between NLRX1, host proteins, and viral components. While the protective features of NLRX1 show promise for an antiviral drug target, the complexity of NLRX1 necessitates a more complete understanding of its mechanisms and actions. We anticipate that further investigation of this under studied sensor will be critical for defining host-pathogen interactions and for future antiviral therapeutic strategies.
